# The Complex Relationship Between Cooling Parameters and Neuroprotection in a Model of Selective Hypothermia

**DOI:** 10.3389/fneur.2022.874701

**Published:** 2022-04-25

**Authors:** Thomas K. Mattingly, Andrew McDavid, Amparo Wolf, Glen Lieber, Ronald Solar, Donald Lee, Stephen P. Lownie

**Affiliations:** ^1^Department of Neurosurgery, University of Rochester, Rochester, NY, United States; ^2^Department of Biostatistics and Computational Biology, University of Rochester, Rochester, NY, United States; ^3^Department of Neurosurgery, Health Sciences North, Sudbury, ON, Canada; ^4^ThermopeutiX, Inc., San Diego, CA, United States; ^5^Department of Medical Imaging, London Health Science Centre, London, ON, Canada; ^6^Division of Neurosurgery, Halifax Infirmary, Halifax, NS, Canada

**Keywords:** therapeutic hypothermia (TH), stroke, ischemia, ischemia/reperfusion injury, model

## Abstract

**Background:**

Hypothermia remains the best studied neuroprotectant. Despite extensive positive large and small animal data, side effects continue to limit human applications. Selective hypothermia is an efficient way of applying neuroprotection to the brain without the systemic complications of global hypothermia. However, optimal depth and duration of therapeutic hypothermia are still unknown. We analyzed a large animal cohort study of selective hypothermia for statistical relationships between depth or duration of hypothermia and the final stroke volume.

**Methods:**

A cohort of 30 swine stroke subjects provided the dataset for normothermic and selective hypothermic animals. Hypothermic parameters including duration, temperature nadir, and an Area Under the Curve measurement for 34 and 30°C were correlated with the final infarct volumes measured by MRI and histology.

**Results:**

Between group comparisons continue to demonstrate a reduction in infarct volume with selective hypothermia. Histologically-derived infarct volumes were 1.2 mm^3^ smaller in hypothermia-treated pigs (*P* = 0.04) and showed a similar, but non-significant reduction in MRI (*P* = 0.15). However, within the selective hypothermia group, more intense cooling, as measured through increased AUC 34 and decreased temperature nadir was associated with larger infarct proportions by MRI [Pearson's *r* = 0.48 (*p* = 0.05) and *r* = −0.59 (*p* = 0.01), respectively]. Reevaluation of the entire cohort with quadratic regression demonstrated a U-shaped pattern, wherein the average infarct proportion was minimized at 515 degree-minutes (AUC34) of cooling, and increased thereafter. In a single case of direct brain tissue oxygen monitoring during selective hypothermia, brain tissue oxygen strongly correlated with brain temperature reduction over the course of selective hypothermia to 23°C.

**Conclusions:**

In a large animal model of selective hypothermia applied to focal ischemia, there is a non-monotone relationship between duration and depth of hypothermia and stroke volume reduction. This suggests a limit to depth or duration of selective hypothermia for optimal neuroprotection. Further research is required to delineate more precise depth and duration limits for selective hypothermia.

## Introduction

While hypothermia remains the best studied neuroprotectant, well-known side effects continue to limit application. Whole body hypothermia is widely practiced in cardiac and neurological surgery, and during critical care for both global and focal ischemia. Selective hypothermia applied only to the region of interest offers an efficient method of neuroprotection without the systemic complications associated with global hypothermia. When combined with modern endovascular technology, selective hypothermia provides the depth needed to reduce CMRO2 within a time frame to potentially impact focal cerebral ischemia e.g., during stroke (1–3).

Uncertainty remains concerning the optimal duration and depth of hypothermia for neuroprotection (4). Human experience favors the use of deep hypothermia, large animal models suggest more moderate levels, but a modern meta-analysis found no advantage between depths of hypothermia (5). An evaluation of endovascular selective hypothermia in a swine model was recently completed (3). This is the largest number of large animals subjected to focal cerebral ischemia with or without focal hypothermic neuroprotection. We analyzed the hypothermia group for a statistical relationship between the depth or duration of hypothermia and ultimate stroke volume.

## Methods

Utilizing a cohort of porcine stroke subjects, we examined the relationships between hypothermia variables and infarct volume. Portions of this cohort including the techniques for producing endovascular hypothermia have been previously reported (3). Following that publication of 13 controls and 12 hypothermia subjects, an additional five hypothermia experiments were performed under the same research protocol (Schulich School of Medicine and Dentistry Animal Use Protocol 2009-079). Analysis of the entire 30 animal cohort for measures of infarction on MRI [ratio of T2 signal abnormality volume to hemispheric volume expressed as a percentage (MRI%)], and pathological volumes of ischemic change (determined by H&E staining) (Path)was performed. The neuroradiology and neuropathology teams remained blinded to the intervention.

**Figure 1:**
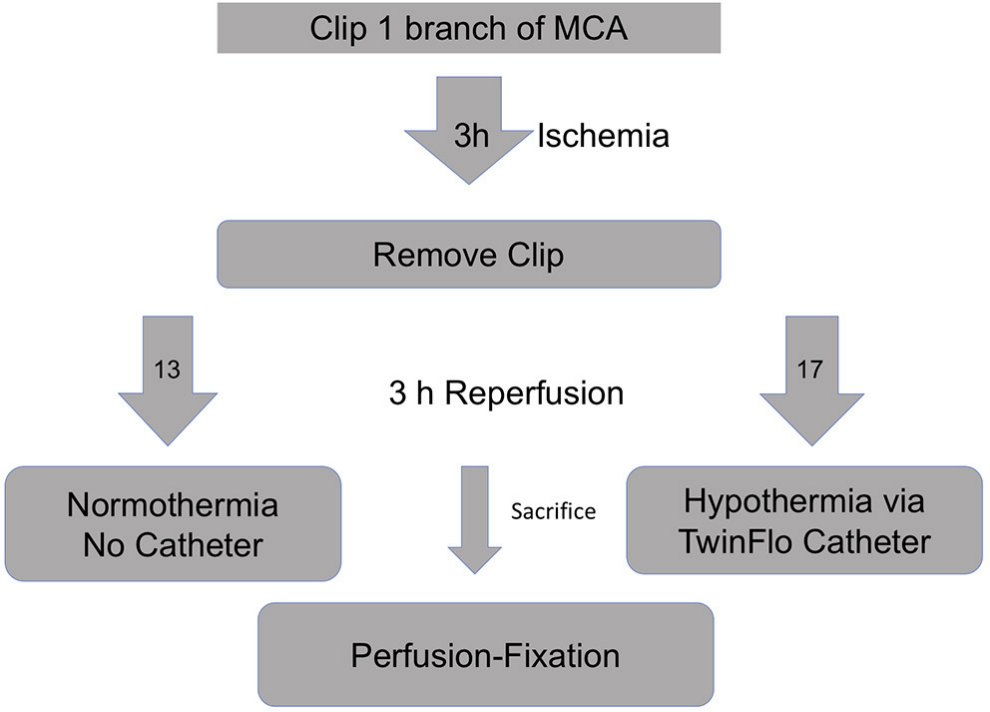
Schematic of the experiment.

Briefly, 50 kg swine were subjected to right craniotomy and temporary clipping of one MCA branch (Figure 1). After 3 h of ischemia, the clip was removed to provide reperfusion. The animals were divided into two groups—selective hypothermia (intervention), and controls (no intervention). For the intervention arm, a purely percutaneous catheter-based hypothermia circuit was assembled. Using a transfemoral approach, the 14F Outer Flow Lumen (OFL) of the TwinFlo catheter (ThermopeutiX, Inc, San Diego, CA) was placed in the descending aorta. The 9.5F Inner Flow Lumen (IFL) was coaxially navigated into the ipsilateral right common carotid artery (CCA). The animal was heparinized, and a standard cardiopulmonary bypass was attached to the OFL (outflow) and IFL (inflow) and purged of air. The bypass was started, and the balloon of the IFL inflated to the point of occlusion such that the R CCA pressure and flow were controlled by the perfusionist. Circuit perfusion was continued until 6 h after the onset of ischemia, at which time the experiment was terminated and the animal killed. For the control arm, the animals were supported under anesthesia without catheterization until 6 h after the onset of ischemia. Postmortem formalin-infused brains were evaluated with MRI the following day using T1 and T2 weighted sequences and a ratio of T2 signal abnormality volume to the hemispheric volume was calculated (=MRI%). Pathologic evaluation of H&E-stained brain slices determined ischemic volumes (cm^3^). We reported two pathology volumes, a total of confluent and scattered stroke volumes (Total Path) and largest single confluent stroke volume excluding scattered stroke (Largest Path). Core (rectal and esophageal), ipsilateral and contralateral nasopharyngeal (brain) temperatures, hemodynamic data, arterial blood gases (ABG), and activated clotting times (ACT) were recorded.

For one of the final hypothermia animals, in addition to rectal and nasopharyngeal temperatures, direct brain temperature monitoring was performed. This had been avoided initially because of concern for hemorrhage during the anticoagulation required for the endovascular procedure, and a trial with a subdural probe yielded temperatures within 2°C of the nasopharyngeal probe. A Licox (Integra, Plainsboro, NJ) brain tissue oxygen and temperature monitor was placed in the contralateral (non-ischemic, no selective-hypothermia) hemisphere. Brain tissue temperature and oxygen tension were successfully recorded for this single experiment.

Comparison of stroke volumes and MRI % between normothermic and hypothermic cohorts was performed using the unpaired *t*-test with Welch's correction. We then examined the relationship between various hypothermia parameters and the infarct volumes within the hypothermia cohort. For the hypothermia parameters we used the duration of cooling (minutes), the lowest temperature achieved (nadir, °C), and a measure combining the time and degree of cooling below a threshold temperature, the Area Under the Curve (AUC-x). This is the region bounded below a fixed temperature intercept, and above the time-temperature curve, so strictly speaking is an “area above the curve,” but we refer to it as an AUC for consistency with previous reports. The AUC represents a “dose” of hypothermia and has been used to describe effectiveness in cooling in prior clinical trials (6). For the hypothermic cohort, temperatures were recorded every 5 min, for up to 3 h (180 min). We took all the temperature measurements below a chosen threshold and summed them to yield a total AUC in degree-minutes for each of the 17 hypothermia animals. We chose cooling below 34°C (AUC 34) as a benchmark based on existing clinical hypothermia data, but we also wanted to examine the effect of cooling below 30°C (AUC30), which this experiment readily achieved.


$$\begin{array}{l} •\;AUC_{34}=5\overset{36}{\underset{1}{\sum }}\max\left(34-T_{i},\;0\right), \end{array}$$

$$\begin{array}{l} •\;AUC_{30}=5\overset{36}{\underset{1}{\sum }}\max\left(30-T_{i},\;0\right), \end{array}$$

where *T*_*i*_ is the temperature in degrees Celsius at the *i*th 5-min interval. Control animals therefore have an AUC of 0.

### Statistical Analysis

Pearson correlations between cooling parameters and infarct volumes within the hypothermia cohort were performed. Correlations were tested by transforming with Fisher's *z*. Statistical significance was set at *p* < 0.05. No adjustments for multiple testing were made.

Based on within group heterogeneous relationships between hypothermia and stroke volume, exploration with scatter plots suggested a non-monotone *U*-shaped relationship could exist. This was tested with quadratic regression, fit with ordinary least squares of the form *E*(*Y*|*x*) = a + bx + cx^2^, where *Y* represents an outcome and *x* is a cooling parameter. This specification allows us to quantify the change in outcome comparing an untreated pig (*x* = 0) with a pig that received one unit treatment with the *b*-coefficient, determine if a *U*-shaped, upside down *U*-shaped or linear relationship is present with the *c*-coefficient, which determines the concavity, and estimate the value of *x* that would lead to minimal injury., given by the value *x* such that 2*cx* + *b* = 0, when *c* > 0. The ordinary non-parametric bootstrap with studentized confidence intervals derived 95% confidence intervals on all these quantities. Data were analyzed using R 3.6.1.

## Results

### Relationship Between Selective Hypothermia Variables and Ischemic Volumes

The final cohort of 13 controls and 17 hypothermia animals continued to demonstrate a 2-fold reduction in every measure of stroke volume in favor of the hypothermia cohort (Table 1). The reduction in total pathology volume was statistically significant, with similar trends in MRI and largest pathology volume. Cooling parameters for the 17 hypothermia animals show that the experiment routinely achieved moderate hypothermia (which we defined as <30°C) in all cases, with temperature nadir near deep hypothermia (median 23.7°C) (Table 2). Times to achieve this level of hypothermia were categorized. Setup time is the time from reperfusion to get the catheters into position, connect and deair the perfusion apparatus, and begin perfusion of cooled blood. Cooling time is the total time during which cooled blood was perfused into the ipsilateral carotid. Cooling time to drop below the threshold of 30°C was evaluated both from the start of cooling (Time to <30°C) and from the start of setup (Total time to <30°C = Time to <30°C + Setup time).

**Table 1:** Summary statistics of neurological injury in control and hypothermia-treated pigs.

**Variable**	**Control**	**Hypothermia**	**P value**
MRI %	0.05 (0.00–0.08)	0.00 (0.00–0.04)	0.15
Total path	1.89 (0.96–2.62)	0.76 (0.04–1.30)	0.04^*^
Largest path	1.03 (0.56–1.47)	0.47(0.04–0.72)	0.07

*Values represent the median and interquartile range. MRI % = ratio of volume of T2 signal abnormality (stroke) to the volume of the cerebral hemisphere. Total path = total stroke volume on H&E stain including confluent and scattered stroke. Largest path = largest confluent stroke volume on H&E stain. P-value from an unpaired t-test with Welch's correction*.

* *Significance at P < 0.05*.

**Table 2:** Selective hypothermia parameters achieved in 17 animals.

	**Median (Q1, Q3)**	**Min, Max**
Setup time (min)	43.0 (33.5, 66.0)	22, 144
Time to <30°C	15.0 (12.0, 35.0)	6, 65
Total cooling time (min)	132.0 (92.5, 140.5)	36, 150
Total time to <30°C	73.0 (49.5–94.0)	45, 155
Temp nadir (°C)	23.7 (22.2, 25.3)	20.3, 27.5

Within the hypothermia group, correlations between the hypothermia “dose” as represented by the AUC below 34°C and below 30°C, and components such as cooling duration and temperature nadir are demonstrated in Table 3. Of note are the positive correlations between stroke volumes (either measured by Pathology or MRI) and the AUC measures or total cooling time. There was also a negative correlation between Temperature nadir and stroke volumes measured either by Pathology or MRI. Correlations reached statistical significance for the relationship between MRI% and AUC 34 and, MRI% and Temperature Nadir. In other words, the more cooling, the larger the stroke volumes.

**Table 3:** Pearson correlations between cooling parameters and outcomes on 17 hypothermia treated animals.

	**Total path**	**Largest path**	**MRI %**
AUC30	0.18	0.08	0.41
AUC34	0.21	0.17	0.48^*^
Cooling time	0.09	0.02	0.36
Temp nadir	−0.28	−0.07	−0.60^*^
IFL MAP	0.24	0.16	0.40
IFL Mean Flow Rate	−0.03	−0.01	−0.26

* *p < 0.05. Correlations were tested by transforming with Fisher's z. No adjustment for multiple testing was made. AUC30 = area under the curve for 30°C. AUC 34 = area under the curve for 34°C. Temp nadir is the lowest temperature achieved averaged in a given experiment. IFL, inner flow lumen; MAP, mean arterial pressure*.

**Figure 2:**
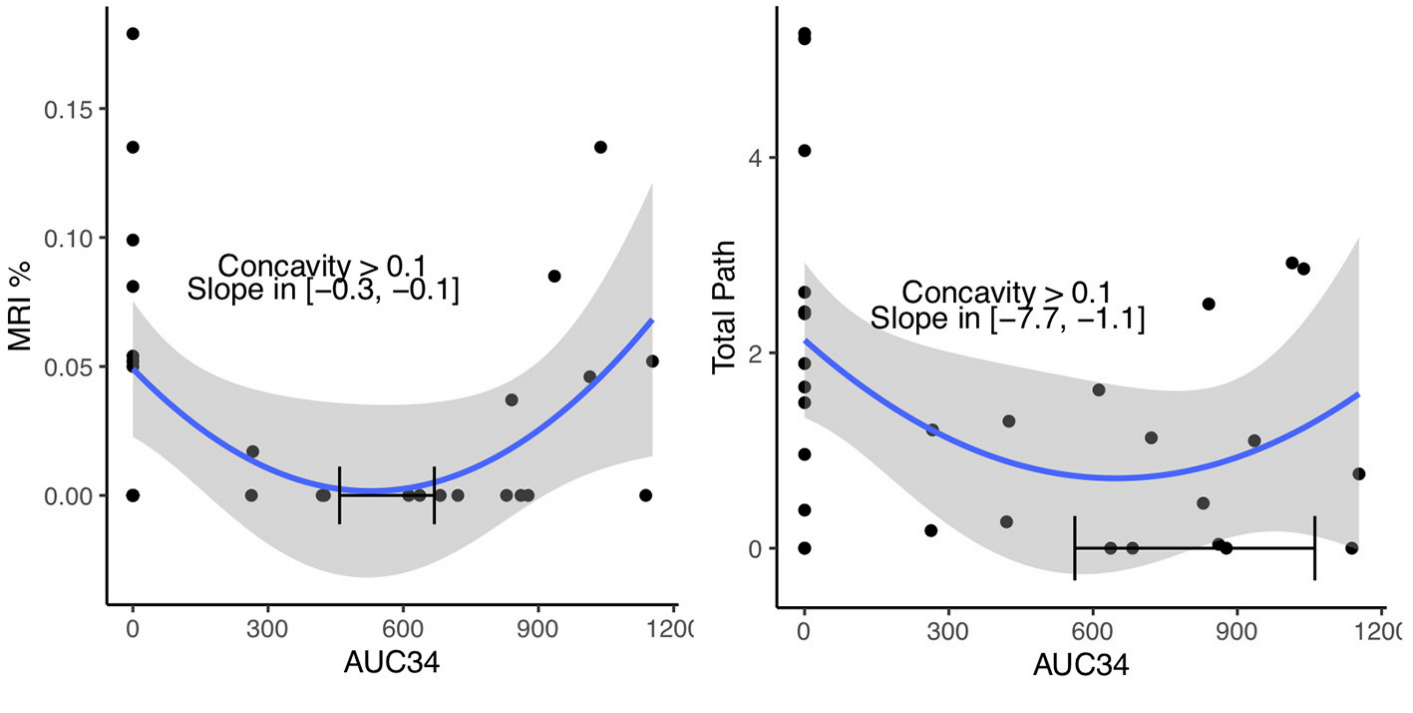
Quadratic regressions of MRI and Total Path vs. AUC34. The relationship estimated using all 30 pigs and confidence region (blue line and gray band) is shown. A 95% confidence interval of the minimizing AUC34, lower 2.5% confidence bound on the concavity and the 95% confidence interval on the slope at AUC34 = 0 are shown on each plot.

This paradoxical result led to reevaluation of the entire cohort (controls and hypothermia) with correlation and scatter plots (Table 4). Grouping the controls (AUC 34 = 0, AUC 30 = 0, cooling time = 0, temperature nadir > 37°C) with the hypothermia cohort caused the sign of many of these correlations to reverse, and is now in the expected direction, with cooling showing a protective effect, suggesting a *U*-shaped relationship. This is demonstrated in AUC 34 vs. MRI, and AUC 34 vs. Total Pathology (Figure 2). Both MRI and Total Path showed statistical evidence of *U*-shaped relationships, with the minimizing AUC34 occurring within the observed range of the data, the lower 2.5% confidence interval on the *c* coefficient, measuring the concavity larger than 0.1, and statistically significant reductions in stroke volume associated with an incremental increase in cooling at the origin (AUC34 = 0). MRI had the most precisely estimated minimizing value, contained in the interval (450, 650) with 95% confidence, while the minimizing value for Total Path was larger and contained in a wider interval.

**Table 4:** Pearson correlations between cooling parameters and outcomes on all 30 animals.

	**Total path**	**Largest path**	**MRI %**
AUC30	−0.27	−0.25	−0.08
AUC34	−0.31	−0.26	−0.11
Cooling time (min)	−0.37^*^	−0.33	−0.19
Temp nadir (deg C)	0.37^*^	0.34	0.21

* *p < 0.05. Correlations were tested by transforming with Fisher's z. No adjustment for multiple testing was made. AUC30 = area under the curve for 30°C. AUC 34 = area under the curve for 34°C. Temp nadir = lowest temperature achieved averaged over the series. Control animals have characteristics of AUC34 = 0, AUC30 = 0, cooling time = 0, temp nadir = 37°C*.

### Relationship Between Direct Brain Temperature and Tissue Oxygen

**Figure 3:**
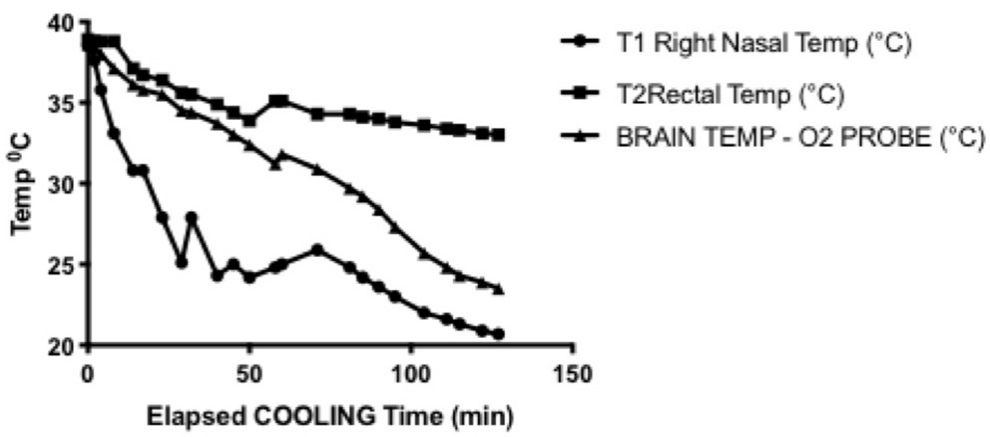
Representative cooling curve for the brain temperature/oxygen measurement selective hypothermia experiment. Right nasal probe was ipsilateral to cooled hemisphere and was used in all experiments as a surrogate for brain temperature. Rectal temperature was core temperature and declines but to a manageable amount. Brain temperature measured through intraparenchymal probe placed in the left hemisphere, demonstrating moderate hypothermia is achieved in the contralateral hemisphere.

**Figure 4:**
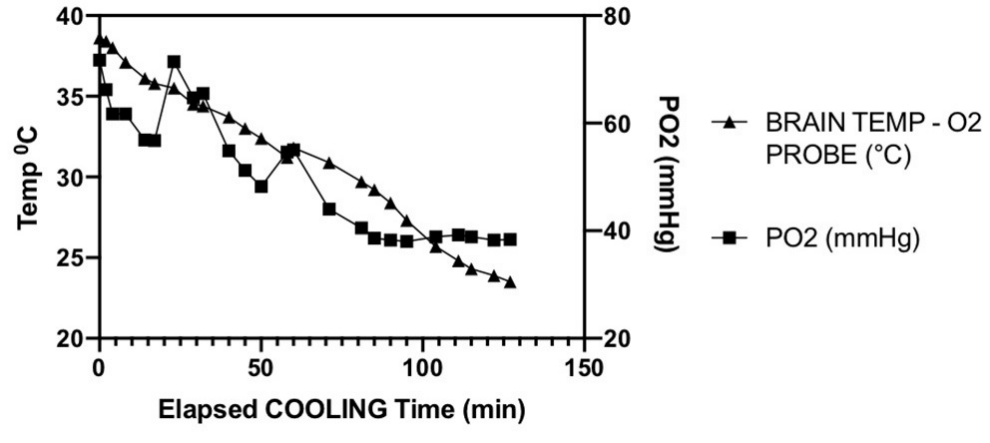
Parallel decline in parenchymal temperature and tissue oxygen in a single animal opposite to the side of ischemia. Pearson R = 0.90.

The single experiment utilizing direct intraparenchymal brain temperature monitoring on the contralateral hemisphere provides a window into selective hypothermic blood perfusion over about 2 h (Figure 3). The ipsilateral surrogate brain temperature (T1 Right nasal temp) descended to 20.7°C during selective hypothermic perfusion into the right hemisphere, while the contralateral non-perfused temperature (measured directly via a Licox monitor- Brain Temp-O2 probe) descended to 23.5°C. Core temperature only dropped to 33°C. The pO2 measurements dropped about 50%, correlating strongly with brain parenchymal temperature (Pearson *r* = 0.90) (Figure 4).

## Discussion

Hypothermia has shown benefit in both global and focal ischemia (5). In focal ischemia, the complications of whole body hypothermia such as cardiac arrhythmia and coagulopathy become quite obvious. One option is to limit cooling to mild hypothermia (33–35°C), which avoids cardiac arrhythmia. However, large animal data suggests hypothermia benefits only when cerebral metabolic rate of oxygen (CMRO2) is reduced significantly, e.g., moderate hypothermia (<25–28°C). The other option is hypothermic cardiopulmonary bypass, which provides deep hypothermia, but is complex, morbid, and relatively inefficient for a focal ischemic lesion. Alternatively, selective hypothermia can achieve moderate-deep hypothermia rapidly, within minutes, while maintaining core temperatures at physiologically acceptable levels. For focal ischemia such as stroke, this is a more efficient and practical solution.

This paper presents a continuation of the largest animal study to evaluate the effect of catheter-based selective hypothermia in focal ischemia. We evaluated both the largest single area of ischemia seen on slices as well as a total ischemia volume in order to distinguish embolic stroke from the endovascular procedure from a contiguous territorial infarct caused by the vessel occlusion. With five additional hypothermic animals, total pathologic volume became statistically significant while the MRI percent hemispheric stroke volume also trended toward benefit. The overall stroke reduction by all measures remained almost 2-fold with the application of selective hypothermic perfusion.

The primary focus was to evaluate the hypothermic cohort for factors that contribute to effective neuroprotection. The impact of time and depth of hypothermia remain uncertain. This large cohort of 17 gyrencephalic animals with rapid achievement of moderate hypothermia (<30°C) provided an opportunity to evaluate these factors. Using specific measures for depth and time, we were unable to find a linear correlation with stroke volume reduction. Instead, within the hypothermia group, some of the animals most effectively cooled had larger strokes. This seems contradictory, but when the controls and hypothermia are analyzed together, the effectiveness of moderate hypothermia appears to have a limit. While this has been noted in cardiac surgery using global hypothermia to prevent global ischemia, this is the first time it has been shown in selective hypothermia (7, 8). There is also small animal data to suggest that cooling below 34°C in a reperfusion model with 90 min ischemia is less helpful (9). It should be noted that small animal hypothermia utilizes whole body cooling, which is distinct from the selective brain cooling that we describe. Furthermore specific to the small animal study, adjustments were made to the depth of hypothermia only. In our series, we had variable depth and length of hypothermia so we evaluate a “dose” of hypothermia. In our series, the best evidence of a *U*-shape curve came with cooling below 34°C, not with cooling below 30°C. Given that the median temperature nadir was well-below 30°C for all hypothermia animals, this may be due to outliers that blunt the effect of better cooling, rather than a suggestion that less cooling is better.

At this point we can only speculate on why moderate selective hypothermia may have this paradoxical effect. One hypothesis is overperfusion, based on an early human series of selective hypothermia (10). In the current series, the TwinFlo catheter allows measurement of distal outflow pressures, which were appropriate in cases where they were recorded, usually 60–70 mm Hg. Inflow rates were also measured, and kept at or below the 300 ml/s rates estimated for normal human common carotid flow rates (1). Finally there was no significant correlation between inflow rate or mean inflow arterial pressure (measured at the tip of the balloon). Thus it seems unlikely that overperfusion is the explanation. Another possibility is inadequate anticoagulation. Heparinization is routine in endovascular procedures, particularly in those that are longer. ACT (Activated clotting time) was measured at least once during the selective hypothermia procedure. However, it is possible that the longer cooling times have lower ACTs late in the cooling, contributing to a larger stroke volume. This will need to be evaluated more carefully in further experiments. Finally, recent cell culture work has demonstrated a differential effect of therapeutic hypothermia on different cell types (neurons, astrocytes, endothelium) within the neurovascular unit (11). From this work, it appears that the depth and duration of therapeutic hypothermia may need to be a function of the time delay in institution in a postreperfusion model. Furthermore, there may be hypothermia-mediated inhibitory effects on neurovascular unit protective interactions. Thus the *U*-shaped effect on stroke volume may not be so surprising.

Finally, we present interesting data of a single intraparenchymal temperature-oxygen probe placed in the contralateral hemisphere. It has been previously shown that contralateral temperatures decrease with unilateral selective hypothermic perfusion (3, 12, 13). Here we confirm that finding, and find a strong correlation with declining oxygen tension. Brain tissue oxygen levels (pBtO2) appears to level off as temperatures drop below 26°C. This finding deserves further exploration with concurrent jugular venous oxygen saturation to determine whether this finding is due to declining cerebral blood flow or decreases in brain oxygen metabolism. A human case report reported pBtO2 increased then decreased during CPB hypothermia followed by deep hypothermic cardiac arrest (14). In TBI, there are reports of decreasing pBtO_2_ with mild hypothermia (32–35°C) (15, 16). Due to mild hypothermia being practiced more widely in humans in the ICU, there is very little data on direct brain tissue oxygen levels in moderate—deep hypothermia, and none utilizing selective hypothermia. GIven the non-monotone effects of moderate hypothermia seen in our experiment, understanding brain tissue metabolism at these temperatures becomes important.

This experiment was designed to reflect real world conditions, as the ischemic insult is 3 h prior to even starting the setup, and just over 4 h before moderate hypothermia (<30°C)is achieved, and even so demonstrates a significant benefit. Mechanical thrombectomy can be performed through the TwinFlo device which has a 510K approval from the FDA. However the logistics of a cardiopulmonary bypass and a large bore endovascular delivery system require careful evaluation before human application to acute ischemic stroke. The potential benefit of transient selective hypothermia in humans is supported by a sizable controlled study of large animal stroke model that achieved a statistically significant reduction in stroke volume.

## Conclusion

While hypothermia remains a proven neuroprotectant, the relationship between moderate hypothermia and stroke volume reduction is non-linear. The technique of selective hypothermia in this large animal model provided rapid onset of moderate hypothermia and demonstrated neuroprotective effect. However, a more profound “dose” of hypothermia did not always yield a lower stroke volume. This significant finding suggests that there may be a “floor” to the effectiveness of hypothermia, even while avoiding the complications of whole-body cooling. This finding deserves more investigation to provide insight into how to maximize the potential of selective brain cooling.

## Data Availability Statement

The original contributions presented in the study are included in the article/supplementary material, further inquiries can be directed to the corresponding author/s.

## Ethics Statement

The animal study was reviewed and approved by Schulich School of Medicine and Dentistry Animal Use Protocol 2009-079, Western University.

## Author Contributions

TM conception and design of the study and wrote the first draft of the manuscript. AW, TM, DL, GL, and RS data collection and analysis. AM performed the statistical analysis and edited manuscript. SL funding support and supervision. All authors approved the final manuscript.

## Funding

This work was supported by Heart and Stroke Foundation, Ontario.

## Conflict of Interest

GL and RS are employed by ThermopeutiX, Inc., and provided the catheters and collected some data. The remaining authors declare that the research was conducted in the absence of any commercial or financial relationships that could be construed as a potential conflict of interest.

## Publisher's Note

All claims expressed in this article are solely those of the authors and do not necessarily represent those of their affiliated organizations, or those of the publisher, the editors and the reviewers. Any product that may be evaluated in this article, or claim that may be made by its manufacturer, is not guaranteed or endorsed by the publisher.
